# The Target Residence Time of Antihistamines Determines Their Antagonism of the G Protein-Coupled Histamine H1 Receptor

**DOI:** 10.3389/fphar.2017.00667

**Published:** 2017-09-25

**Authors:** Reggie Bosma, Gesa Witt, Lea A. I. Vaas, Ivana Josimovic, Philip Gribbon, Henry F. Vischer, Sheraz Gul, Rob Leurs

**Affiliations:** ^1^Amsterdam Institute for Molecules, Medicines and Systems, Division of Medicinal Chemistry, Faculty of Science, VU University Amsterdam Amsterdam, Netherlands; ^2^Fraunhofer Institute for Molecular Biology and Applied Ecology Screening Port Hamburg, Germany

**Keywords:** residence time, recovery time, GPCR, histamine H1 receptor, calcium mobilization, DMR, ligand binding kinetics, insurmountable antagonism

## Abstract

The pharmacodynamics of drug-candidates is often optimized by metrics that describe target binding (K_d_ or K_i_ value) or target modulation (IC_50_). However, these metrics are determined at equilibrium conditions, and consequently information regarding the onset and offset of target engagement and modulation is lost. Drug-target residence time is a measure for the lifetime of the drug-target complex, which has recently been receiving considerable interest, as target residence time is shown to have prognostic value for the *in vivo* efficacy of several drugs. In this study, we have investigated the relation between the increased residence time of antihistamines at the histamine H_1_ receptor (H_1_R) and the duration of effective target-inhibition by these antagonists. Hela cells, endogenously expressing low levels of the H_1_R, were incubated with a series of antihistamines and dissociation was initiated by washing away the unbound antihistamines. Using a calcium-sensitive fluorescent dye and a label free, dynamic mass redistribution based assay, functional recovery of the H_1_R responsiveness was measured by stimulating the cells with histamine over time, and the recovery was quantified as the *receptor recovery time*. Using these assays, we determined that the receptor recovery time for a set of antihistamines differed more than 40-fold and was highly correlated to their H_1_R residence times, as determined with competitive radioligand binding experiments to the H_1_R in a cell homogenate. Thus, the receptor recovery time is proposed as a cell-based and physiologically relevant metric for the lead optimization of G protein-coupled receptor antagonists, like the H_1_R antagonists. Both, label-free or real-time, classical signaling assays allow an efficient and physiologically relevant determination of kinetic properties of drug molecules.

## Introduction

Despite the wide utilization of binding affinity and related drug binding metrics in drug discovery, it is increasingly debated whether these values provide sufficient information to allow prediction of *in vivo* efficacy (Waring et al., 2015). It is currently recognized that the binding rate constants of drug-target interactions may bring additional prognostic value in scenarios, in which there are no stable ligand concentrations, as is typical for *in vivo* systems (Swinney, 2004; Copeland et al., 2006; Lu and Tonge, 2010; Guo et al., 2014; Hoffmann et al., 2015). In particular the target residence time, which is the reciprocal of the dissociation rate constant of a bound ligand, is thought to be an important metric for drug optimization. A long residence time is, for example, related to an insurmountable mode of antagonism when endogenous agonist concentrations are transiently increased, as is observed in the case of neuronal signaling (Vauquelin et al., 2012). Additionally, compounds with a long residence time show prolonged drug-target occupancies beyond the point at which pharmacologically active drug concentrations are present in the blood (Ramsey et al., 2011; Bradshaw et al., 2015). A long residence time (>1 h) was, e.g., observed for several clinically used antihistamines that bind to the histamine H_1_ receptor (H_1_R), an important drug target for the treatment of, e.g., allergic rhinitis (Anthes et al., 2002; Gillard et al., 2002; Slack et al., 2011b; Bosma et al., 2016). In analogy with insurmountable antagonism observed in neuronal signaling, it has been described that histamine levels after allergen challenge are only transiently increased, implying that an insurmountable mode of antagonism could effectively block high concentrations of histamine (Petersen et al., 1996). Moreover, in an *ex vivo* organ bath experiment in which antagonists were continuously removed, it was observed that the long residence time compounds azelastine and GSK1004723 both retained a long inhibition of the histamine-induced, H_1_R mediated bronchial contraction (Slack et al., 2011a,b). *In vivo*, the clinically used anthistamine levocetirizine with a long residence time, also shows hysteresis of efficacy after depletion of free levocetirizine concentrations in the blood (Gillard et al., 2002; Schoepke et al., 2013). Together, these data suggest that residence time is an important predictor of *in vivo* efficacy of H_1_R antihistamines.

The H_1_R is a prototypic member of the therapeutically relevant family of G protein-coupled receptors (GPCRs). The kinetic binding rate constants of unlabeled ligands for GPCRs are often measured using radioligand or fluorescent binding techniques (Schuetz et al., 2017). In these experiments, the effect of the unlabeled ligand on the binding of the labeled ligand is measured over time and kinetic binding rate constants are determined. Such experiments are often done using cell membranes as a source of the receptor. In this study, we developed methods to measure the kinetics of H_1_R antagonism upon depletion of the free concentration antihistamine, by measuring recovery of the histamine-induced response over time in a physiologically relevant cell system. To this end, a fluorescent based calcium mobilization assay and a label-free, dynamic mass redistribution (DMR) based assay were evaluated for the measurement of histamine-induced responses in human HeLa cells, cervical cancer cells known to endogenously express low levels of the H_1_R (Govoni et al., 2003; Das et al., 2007). Using these assay formats, it is shown that the receptor recovery time is correlated to the residence time of antihistamines, hence, this parameter might therefore have predictive value for the *in vivo* efficacy of such ligands. The described orthogonal assays will also be very relevant for future GPCR drug discovery projects, as both calcium signaling, as well as DMR-responses, can be measured for a large number of GPCRs (Charlton and Vauquelin, 2010; Schröder et al., 2010).

## Materials and Methods

### Materials

Fetal bovine serum (FBS) was from Bodinco (Alkmaar, the Netherlands). Penicillin/streptomycin 100x was purchased from GE healthcare (Uppsala, Sweden). Hank’s balanced salt solution (HBSS), BCA protein assay kit and Fluo-4 NW dye were from Thermo Fisher Scientific (Waltham, MA, United States). A 1x trypsin solution, Dulbecco’s modified medium/Ham’s F-12 (DMEM/F12) and DMEM were from Sigma–Aldrich (St. Louis, MO, United States). Sterile, black and clear bottom 96-well plates were from VWR (Radnor, PA, United States). Polypropylene 384 well microplates were obtained from Greiner Bio-One GmbH (Frickenhausen, Germany). Transfection reagent linear 25 kDa polyethylenimine (PEI) was from Polysciences (Warrington, PA, United States). Branson sonifier 250 homogenizer was from Emerson (St. Louis, MO, United States). From Perkin Elmer (Waltham, MA, United States) the following were obtained: [^3^H]mepyramine; GF/C plates; Microscint-O; the Cell Harvester; the Wallac microbeta; the EnSpire^®^ Multimode Plate Reader equipped with Corning^®^ Epic^®^ Label-free technology; the JANUS MDT Automated Workstation; EnSpire^®^ -LFC 384- fibronectin coated plates. The NOVOstar plate reader was from BMG Labtech (Ortenberg, Germany). Pharmacological tool compounds were acquired from the following commercial sources: probenecid, olopatadine hydrochloride and acrivastine from Sigma–Aldrich (St. Louis, MO, United States); Histamine hydrochloride from TCI chemicals (Portland, OR, United States); mepyramine maleic acid from Research Biochemicals International (Natick, MA, United States); levocetirizine dihydrochloride from Biotrend (Köln, Germany); doxepin hydrochloride and triprolidine hydrochloride from Tocris Bioscience (Bristol, United Kingdom); desloratadine from HaiHang Industry, Co., Ltd (Jinan City, China). HeLa cells and HEK293T cells were from an in-house eukaryotic cell biobank as described in previous publications (Bakker et al., 2004; Bosma et al., 2016). Compounds VUF14454, VUF14493, VUF14506, and VUF14544 were synthesized at the Vrije Universiteit Amsterdam and were fully characterized with respect to purity and identity. All pharmacological compounds were dissolved in DMSO to a stock concentration of 10^-2^ M unless otherwise specified in Section “Materials and Methods.” Moreover, materials from a deviating source are stated explicitly in the respective section of the Methods. All other chemicals are of analytical grade quality.

### Radioligand Binding Experiments

#### Cell Culture

HEK293T cells and HeLa cells were cultured in a humidified atmosphere at 37°C with 5% CO_2_ in DMEM and DMEM/F12 medium respectively which was supplemented with 10% FBS and 1x penicillin/streptomycin. Cell pellets of HEK293T cells transiently expressing the HA-hH_1_R were derived as described before (Bosma et al., 2016). Cell pellets of HeLa cells were derived by flushing cells from a sub confluent 10 cm^2^ dish. Cells were then washed with PBS [137 mM NaCl, 2.7 mM KCl, 10 mM Na_2_HPO_4_ and 2 mM KH_2_PO_4_] and consecutively pelleted by centrifuge steps. Cell pellets were then stored until further experimentation at -20°C.

#### Radioligand Binding

Characterization of [^3^H]mepyramine and unlabeled ligands using radioligand binding experiments was described extensively before with minor changes (Bosma et al., 2016). In short, cell pellets were reconstituted in binding buffer [50 mM Na_2_HPO_4_/KH_2_PO_4_ pH 7.4] and homogenized.

For saturation binding experiments 1–15 nM [^3^H]mepyramine in the absence or presence of 10^-5^ M mianserin was incubated with either 0.5–3 μg cell homogenate of HEK293T cells or 20–60 μg of HeLa cells for 4 h at 37°C. Binding affinity (K_d_) and B_max_ was determined from the total (without mianserin) and non-specific (with mianserin) binding of [^3^H]mepyramine using non-linear regression in Prism 6.0 (GraphPad Software, San Diego, CA, United States). Reported pK_d_ and B_max_ values represent the mean and SEM of ≥3 experiments.

Competition binding experiments were performed by incubating (0.5–3 μg) HEK293T cell homogenate transiently expressing the H_1_R with a single concentration (3–6 nM) [^3^H]mepyramine and increasing concentrations unlabeled ligand (10^-11^ to 10^-4^ M) for 4 h at 37°C. From the resulting radioligand displacement curves, the IC_50_ was determined and binding affinity (K_i_) was then derived by using the Cheng–Prusoff equation (Cheng and Prusoff, 1973). Reported pK_i_ represent the mean and SEM of ≥3 experiments.

Kinetic binding rate constants of [^3^H]mepyramine were determined to be 0.22 min^-1^ (*k*_off_) and 1.1 × 10^8^ M^-1^min^-1^ (*k*_on_) (Bosma et al., 2016). To determine the kinetic binding rate constants of unlabeled ligands (0.5–3 μg) a HEK293T cell homogenate transiently expressing the H_1_R was co-incubated with a single concentration [^3^H]mepyramine 1.5–12 nM for various incubation times (0–81 min) at 25°C in the presence and absence of three concentrations unlabeled ligand. Final concentrations unlabeled ligands were selected to have various levels of inhibition of [^3^H]mepyramine binding within the total incubation time. To have well-separated binding curves over the course of the experiments, ligands with a fast *k*_off_ were used on approximately 1–10 times the K_i_ value whereas the slow dissociating ligands had to be used on approximately 10–100 times the K_i_ value. The resulting binding of [^3^H]mepyramine to the H_1_R was analyzed using the Motulsky–Mahan model yielding the *k*_on_ and *k*_off_ of the unlabeled ligands (Motulsky and Mahan, 1984). Moreover the binding affinity and residence time could be calculated from the binding rate constants, with K_d,calc_ = *k*_off_/k_on_ and residence time (RT) = 1/*k*_off_. Reported *k*_on_, *k*_off_, K_d,calc_, and RT values represent the mean and SEM of ≥3 experiments.

### Histamine-Induced Intracellular Calcium Mobilization

#### Fluorescent Detection of Calcium Mobilization Using Fluo4NW

HeLa cells were cultured as described above. HeLa cells were lifted from a subconfluent dish by incubating with a 1x trypsin solution for 4 min. Cells were then seeded in a black, clear bottom 96-well plate, with 2 × 10^4^ cells per well. Subsequently, the cells were pre-incubated overnight in culture medium with a range of antagonist concentrations or without antagonist, as is specified per assay format below. After 18–20 h, assay buffer was prepared by supplementing HBSS with 20 mM HEPES pH 7.4 and 2.5 mM probenecid (from a 2.5 × 10^-1^ M stock in water). Dye solution was then prepared by dissolving one aliquot of Fluo4 NW in 22 mL assay buffer. Medium was aspirated and Fluo4 NW dye solution was supplemented to the cells and incubated for an hour at 37°C in the presence of the respective antagonist. After labeling the cells with Fluo4 NW, readout of the calcium response was measured per individual well in series. For each measurement, histamine was injected to induce a peak calcium response and consecutively, Triton-X-100 was injected to lyse the cells for a saturated Fluo4 NW calcium response. Fluorescence at λ_excitation_ 494 nm and λ_emission_ 516 nm was measured with the NOVOstar, which was set to 37°C, once per second for three segments: firstly, the background signal was quantified as the average fluorescence before histamine injection (F_b_); secondly, the histamine induced calcium mobilization was quantified as the fluorescent intensity between the histamine injection and Triton-X-100 injection (F_HA_); finally, the saturated calcium response was quantified as the maximum signal after Triton-X-100 injection (F_t_). The histamine-induced response (F_HA_) was then normalized according to Eq. 1.

(1) $$\begin{array}{c} \mathrm{normalized}\mathrm{fluorescent}\mathrm{response}=\frac{F_{\mathrm{HA}}-F_{b}}{F_{t}-F_{b}} \end{array}$$

#### Histamine Dose Response Relationship

Cells were pre-incubated with or without increasing concentrations of the antagonists mepyramine (10^-8.3^ to 10^-6.7^ M), doxepin (10^-8^ to 10^-9.2^ M), olopatadine (10^-9^ to 10^-7.8^ M), or levocetirizine (10^-8.1^ to 10^-6.9^ M). Furthermore, for every concentration antihistamine, cells were stimulated with increasing concentrations histamine (10^-8^ to 10^-3.1^ M with 0.7 log-unit steps; prepared from a 10^-1^ M stock in deionized water) using duplicate wells per experiment. Per well fluorescence was detected for 60 s as described with a 20 μL histamine injection at the 20 s mark and a 50 μL Trition-X-100 (final concentration 1.5%) injection at the 50 s mark. Either the normalized peak fluorescence or the area under the curve (AUC) for 30 s after histamine stimulation was then used as a measure for the calcium mobilization, which was plotted against the respective histamine concentration. The AUC was quantified using GraphPad Prism (GraphPad Software, San Diego, CA, United States). Settings were as such that peaks were ignored when there was less than 1% difference between min and max fluorescence and/or peaks shaped by fewer than 10 data points. The resulting dose-response curves were analyzed by non-linear regression according to Eq. 2.

(2) $$\begin{array}{c} Y=Bottom\;+\;\frac{\mathrm{Top}-\mathrm{Bottom}}{1+10^{\log{\mathrm{EC}}_{50}-\log[\mathrm{histamine}]}} \end{array}$$

#### Receptor Recovery of Calcium Mobilization after Antagonist Washout

One 96-well plate with HeLa cells was separated into four sections and each section was pre-incubated with a respective 10 times K_i_ concentrations of antihistamine or vehicle condition. Hence, three different antihistamines were used per 96-well plate. Following Fluo4 NW loading in the presence of the respective concentration antihistamine, cells were washed and reconstituted in 100 μL assay buffer that was pre-heated to 37°C (**t_0_**). Subsequently, the histamine induced calcium mobilization was measured per well, alternating between the four conditions (pre-treatment with vehicle and each of the three antihistamines), for 24 cycles. The first measurement was started approximately 2 min after washing the cells and subsequent wells were measured with 75 s intervals. Fluorescence was detected during this 75 s as described and 20 μL histamine (final concentration 10^-5^ M) was injected at the 10 s mark and 50 μL Trition-X-100 (final concentration 1.5%) at the 65 s mark. The normalized peak response following histamine stimulation was then plotted against the washout time (t) defined as the time between **t_0_** and histamine injection in the respective well. The data was analyzed using non-linear regression with a one-phase association model in GraphPad Prism according to Eq. 3.

(3) $$\begin{array}{c} {Y=Y}_{0}+(Y_{\max}-Y_{0})\times (1-e^{\left(-k_{\mathrm{rec}}\times t\right)}) \end{array}$$

Here Y_0_ was constrained to be 0, Y_max_ is the histamine-induced response upon reaching a steady-state and k_rec_ is the recovery rate of the histamine induced response. For cells pre-treated with olopatadine, insufficient receptor recovery was observed within the time-frame of the experiment and Y_max_ was therefore constrained to be the average histamine response measured in cells pre-treated with vehicle condition. For all other antihistamines a free fit of Y_max_ was allowed, reported in the results as the steady-state recovery (%). Receptor recovery times (RecT) were calculated for each experiment as the reciprocal of the k_rec_. Reported k_rec_ and RecT values are the mean ± SEM of ≥3 experiments.

### Dynamic Mass Redistribution (DMR)

For the measurement of DMR in HeLa cells upon treatment by agonists and antagonists, a resonant waveguide grating (RWG) biosensor method was used. This is a widely used method for non-invasive quantification of GPCR modulation in living cells and details of the technology are described elsewhere (Fang et al., 2006, 2008).

#### DMR Receptor Recovery after Washout

HeLa cells were grown in DMEM High Glucose medium with L-Glutamine supplemented with 10% FBS and 1x penicillin/streptomycin [all reagents were obtained from Capricorn Scientific GmbH (Ebsdorfergrund, Germany)] and incubated in humidified atmosphere at 37°C and 5% CO_2_ in air.

For DMR experiments, culture medium was transferred to an EnSpire-LFC 384- fibronectin coated plate (10 μl/ well) and incubated for 30 min at room temperature.

Afterward, antagonists were diluted in cell culture medium to a 10x K_i_ final assay concentration and 10 μL per well were transferred to the LFC plate. All compounds were diluted from a 100 mM stock in DMSO, except for desloratadine (25 mM), VUF14454 (2.5 mM), and acrivastine (5 mM). 1.5 × 10^4^ HeLa cells were seeded per well, resulting in a final assay volume of 30 μL/well. As vehicle control wells with medium/DMSO treated cells were included. The LFC plate was incubated overnight in a humidified atmosphere at 37°C and 5% CO_2_ in air. On the next day assay buffer [HBSS (Sigma–Aldrich), 20 mM HEPES (Sigma–Aldrich), 0.5% (v/v) DMSO] was adapted to room temperature before use. 22–24 h after cell seeding antagonists were removed from the LFC plate and wells were washed four times with label-free assay buffer (25 μL/ well). The total assay volume after the washing step was 30 μL/ well.

A dilution series of histamine (from 10^-1^ M stock in deionized water) was prepared in label-free assay buffer and dispensed into an intermediate microplate. In all experiments a DMSO concentration of 0.5% (v/v) was not exceeded. The LFC plate was placed in an EnSpire^®^ Multimode Plate Reader equipped with Corning^®^ Epic^®^ Label-free technology. 1 and 2 h after antagonist washout a baseline was recorded (5 min) and histamine was transferred from the intermediate microplate into the LFC plate using a JANUS^®^ MDT Automated Workstation and a 30 min kinetic DMR measurement was recorded on the EnSpire^®^ Multimode Plate Reader at room temperature.

Data was analyzed individually for each quadrant of the 384 well LFC plate using GraphPad Prism. Peak response values of vehicle-treated cells were plotted against the log_10_ histamine concentration and data was fitted using a four-parameter sigmoidal fit. The maximum (100% effect) and minimum (0% effect) values of the control dose response curve were used to normalize the histamine induced peak responses of antagonist treated cells, to give a relative peak response (%).

The recovery rate (k_rec_) of the histamine induced DMR-response after antagonist washout was estimated using Eq. 3 when the effect was significantly different at both time points (Supplementary Figure 1). For estimation of the recovery rate the response induced by a saturating concentration histamine (60 μM) was used as determined for: vehicle treated cells (Y_max_); antagonist treated cells 1 h after washout (Y_0_); antagonist treated cells 2 h after washout (Y). Depicted values represent mean and standard deviation of two independent experiments. Moreover, for antihistamines that allowed full recovery within 1 h after washing away unbound ligands, RecT was estimated to be <30 min. This estimation is based on a >90% inhibition right after washout (*t* = 0) and a functional recovery of >90% after 1 h.

#### DMR Agonist/Antagonist Co-incubation

An LFC plate was activated with 10 uL culture medium. Afterward, 1.5 × 10^4^ HeLa cells per well were seeded and incubated over night as described above. 22–24 h after cell seeding medium was removed from the LFC plate and wells were washed four times with label-free assay buffer (25 μL/ well). The LFC plate was placed in an EnSpire^®^ Multimode Plate Reader.

A dilution series of antagonist was prepared in label-free assay buffer and dispensed together with histamine (Selleck Chemicals, Munich, Germany) into an intermediate microplate (Polypropylene 384 well microplate).

Two hour after washing a baseline was recorded (10 min) on an EnSpire^®^ Multimode Plate Reader. Afterward, compounds were transferred from the intermediate microplate into the LFC plate and a 60 min kinetic DMR measurement was started.

For data analysis the AUC was determined from 1 to 20 min and plotted against the antagonist concentration. Data was fitted using a sigmoidal four-parameter fit and EC_50_ values were determined.

## Results

It is well-known that the human adenocarcinoma HeLa cell line endogenously expresses functional histamine H_1_Rs, coupled to the mobilization of calcium and changes in cell morphology (Smit et al., 1992; Yu et al., 2006). As such, this cell line was selected as a physiological relevant test model for the proposed kinetic receptor recovery studies. Expression levels of the H_1_R were quantified in HeLa cells as well as in transiently transfected HEK293T cells using a radioligand saturation binding experiment (**Table 1**). Increasing concentrations of the H_1_R antagonist [^3^H]mepyramine were incubated with cell homogenates of the respective cell line for 4 h at 37°C. [^3^H]mepyramine displayed comparable affinity for H_1_R on HeLa cells and HEK293T cells with pK_d_ values of 8.5 ± 0.2 and 8.1 ± 0.1, respectively. A very low window of specific [^3^H]mepyramine binding was observed for the HeLa cell line, in sharp contrast to the HEK293T cells, which transiently expressed the human H_1_R at >100-fold higher expression levels (**Table 1**). The observed expression of the H_1_R on HeLa cells was in the same order of magnitude as described in literature (55–130 fmol/mg protein) (Govoni et al., 2003; Das et al., 2007).

**Table 1:** Expression of the H_1_R and binding affinity of [^3^H]mepyramine.

	H_1_R expression	pK_d_	B_max_ (fmol/mg)
HeLa cells	Endogenous	8.5 ± 0.2	200 ± 130
HEK293T cells	Transient	8.1 ± 0.1	36000 ± 10000

*Average values are shown ±SEM of ≥3 experiments.*

**FIGURE 1:**
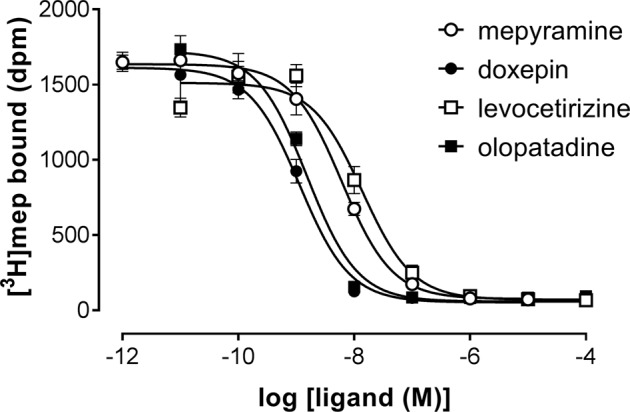
Competition binding for the histamine H_1_ receptor by [^3^H]mepyramine and unlabeled antihistamines. An homogenate of HEK293T cells transiently expressing the H_1_R was incubated with increasing concentrations unlabeled antihistamines and 5.6 nM [^3^H]mepyramine for 4 h at 37°C in 50 mM Na_2_HPO_4_/KH_2_PO_4_ pH 7.4. Representative graphs of ≥3 experiments are shown with mean ± SD of triplicate values (*n* = 3).

A set H_1_R antihistamines was selected based on the diversity in chemotype and expected diversity in receptor residence times (Supplementary Table 1) (Gillard et al., 2002; Bosma et al., 2016). First, the equilibrium (i.e., steady-state) binding of antihistamines to the H_1_R was characterized using radioligand binding experiments. To determine the binding affinity (pK_i_) of antihistamines for the H_1_R, a homogenate of HEK293T cells transiently expressing the H_1_R was co-incubated with a single concentration [^3^H]mepyramine (3–6 nM) and increasing concentrations of unlabeled antihistamines for 4 h at 37°C. Examples of four [^3^H]mepyramine displacement curves for the prototypical H_1_R antihistamines mepyramine, doxepin, levocetirizine, and olopatadine are depicted in **Figure 1**. More than 2 log unit differences in pK_i_ values were observed between antihistamines (**Table 2**) and the respective K_i_-values were comparable to those described previously (Nonaka et al., 1998; Wieland et al., 1999; Gillard et al., 2002; Kuhne et al., 2016).

**Table 2:** Characterization of the binding of antihistamines to the H_1_R using competitive [^3^H]mepyramine binding experiments.

	pK_i_ (37°C)	pK_d,calc_ (25°C)	*k*_on_ (25°C) (10^6^⋅min^-1^⋅M^-1^)	*k*_off_ (25°C) (min^-1^)	RT^a^ (min)
Mepyramine	8.5 ± 0.0	8.8 ± 0.0	200 ± 50	0.28 ± 0.05	3.9 ± 0.6
Levocetirizine	8.1 ± 0.1	8.2 ± 0.1	1.2 ± 0.2	0.008 ± 0.001	140 ± 20
Doxepin	9.3 ± 0.1	9.1 ± 0.1	70 ± 10	0.06 ± 0.02	22 ± 7
Olopatadine	9.1 ± 0.0	8.5 ± 0.0	1.8 ± 0.1	0.006 ± 0.000	170 ± 10
Triprolidine	8.3 ± 0.1	8.1 ± 0.1	36 ± 5	0.30 ± 0.04	3.5 ± 0.4
Acrivastine	7.2 ± 0.0	7.0 ± 0.0	0.6 ± 0.1	0.065 ± 0.004	15.6 ± 0.9
Desloratadine	9.1 ± 0.1	9.5 ± 0.0	25 ± 12	0.008 ± 0.003	160 ± 50
VUF14544	7.6 ± 0.1	7.8 ± 0.1	100 ± 40	1.3 ± 0.3	0.9 ± 0.2
VUF14454	8.2 ± 0.1	8.6 ± 0.0	250 ± 90	0.6 ± 0.1	1.8 ± 0.3
VUF14506	7.7 ± 0.1	7.9 ± 0.0	3.6 ± 0.7	0.05 ± 0.01	22 ± 4
VUF14493	8.3 ± 0.0	8.4 ± 0.1	300 ± 100	0.9 ± 0.2	1.1 ± 0.2

*Average values are shown ±SEM of ≥3 experiments.*

*^a^RT, residence time (1/*k*_*off*_).*

**FIGURE 2:**
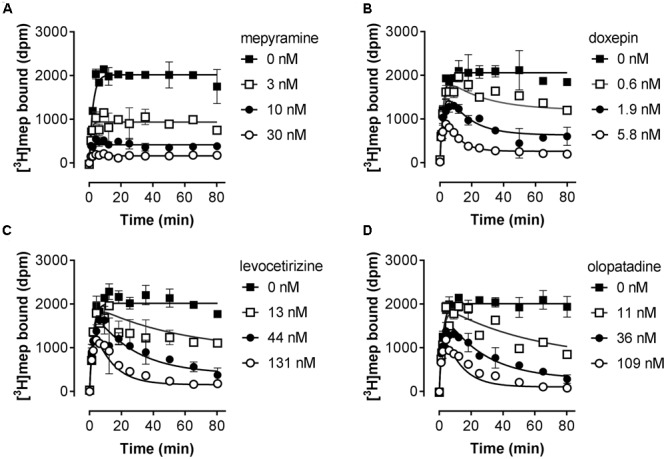
Competitive association of [^3^H]mepyramine and unlabeled antihistamines to the H_1_R. An homogenate of HEK293T cells transiently expressing the H_1_R was incubated with a 2.4 nM concentration [^3^H]mepyramine in the absence or presence of 1 to 100-fold K_i_ concentrations of the respective unlabeled antihistamines for the indicated time at 25°C in 50 mM Na_2_HPO_4_/KH_2_PO_4_ pH 7.4. Results are shown for the unlabeled antihistamines mepyramine **(A)**, doxepin **(B)**, levocetirizine **(C)** and olopatadine **(D)**. Representative graphs of ≥3 experiments are shown with mean ± SD of duplicate values (*n* = 2).

Subsequently, the kinetic binding rate constants of antihistamines were determined in [^3^H]mepyramine competitive association experiments. Antihistamines were therefore co-incubated with [^3^H]mepyramine and a homogenate of HEK293T cells, transiently expressing the human H_1_R, for 0–81 min at 25°C (**Figure 2** and **Table 2**). In such competitive association binding experiments a typical overshoot pattern in the binding of the radioligand is observed when it has a higher *k*_off_ than the unlabeled ligand at the receptor (Motulsky and Mahan, 1984). In the presence of unlabeled mepyramine (**Figure 2A**) there is no overshoot pattern in the binding of the radioligand to the H_1_R, indicating that the ligand *k*_off_ at the H_1_R is similar (or higher) for mepyramine as compared to [^3^H]mepyramine. In the presence of doxepin (**Figure 2B**), levocetirizine (**Figure 2C**), and olopatadine (**Figure 2D**) an overshoot in [^3^H]mepyramine binding to the H_1_R is observed which reflects the relative low *k*_off_ of these unlabeled ligands at the H_1_R compared to the *k*_off_ of [^3^H]mepyramine. The kinetic binding rate constants (*k*_on_ and *k*_off_) are determined from these kinetic binding traces using the Motulsky–Mahan model (Motulsky and Mahan, 1984). The representative antihistamines mepyramine, doxepin, levocetirizine and olopatadine, depicted in **Figures 2A–D**, illustrate the range in *k*_off_ values that were obtained from competitive association experiments. From the kinetic binding constants, the respective binding affinity for the H_1_R could be calculated [pK_d,calc_ = -log(*k*_off_/*k*_on_)]. Additionally, the residence time was calculated (RT = 1/*k*_off_), which is a measure for the length of H_1_R engagement by the antihistamine. The binding characteristics of all tested ligands are reported in **Table 2**.

Based on the kinetic and equilibrium [^3^H]mepyramine binding experiments, it is shown that the tested set of antihistamines display >100-fold differences in K_i_, binding rates and residence times for their binding to the H_1_R (**Table 2**). The >35-fold differences in residence time at the H_1_R between levocetirizine (RT = 140 min), desloratadine (RT = 160 min) and mepyramine (RT = 3.9 min) agreed with previously reported data showing a 50 to 140-fold longer residence time for levocetirizine and desloratadine compared to mepyramine (RT, mepyramine: 0.8–8 min) (Gillard et al., 2002; Gillard and Chatelain, 2006; Schiele et al., 2015). Interestingly, the differences in residence time at the H_1_R between antihistamines were not reflected by the respective differences in the pK_i_. For example, levocetirizine has a similar H_1_R binding affinity but much longer residence time at the H_1_R as compared to mepyramine (**Table 2**).

**FIGURE 3:**
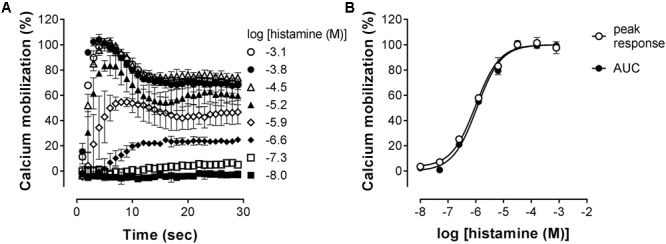
Histamine-induced intracellular calcium mobilization in HeLa cells endogenously expressing the H_1_R. **(A)** Calcium dependent fluorescence was measured over time after injection of increasing histamine concentrations at *t* = 0. Representative graphs are shown of ≥3 experiments depicting the mean ± SD of duplicate values (*n* = 2). **(B)** For comparison, peak response and area under the curve (AUC) were determined from the kinetic calcium mobilization traces and plotted against the histamine concentration.

The H_1_R is known to activate Gαα_q_, leading to increased intracellular calcium levels via activation of phospholipase C (Jacob et al., 1988; Smit et al., 1992). Indeed, in HeLa cells, an histamine-induced dose dependent increase in the calcium mobilization can be measured as an increase in the fluorescence of the calcium-sensitive Fluo4 NW dye (λ_excitation_ 494 nm and λ_emission_ 516 nm). As can be seen in **Figure 3A**, a peak increase in fluorescence is obtained within seconds after stimulation of HeLa cells with histamine. The fluorescence signals then decrease to a steady-state level for at least 30 s. The H_1_R response was quantified both by the histamine-induced peak response or the AUC observed between 0 and 30 s after stimulating with histamine. Plotting the log concentration histamine versus the relative calcium mobilization leads to overlapping histamine-dose-response curves. Analyzing both curves with a four-parameter sigmoidal fit resulted in similar pEC_50_ values of 6.1 ± 0.1 and a Hill slope of 1.1 ± 0.1 (**Figure 3B**).

**FIGURE 4:**
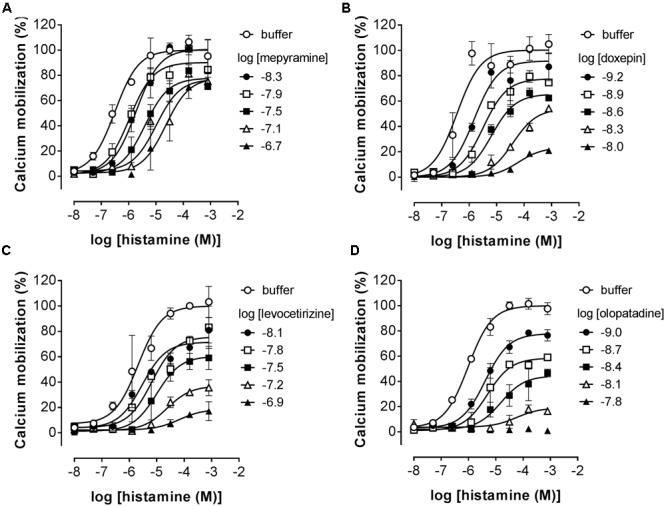
Insurmountable antagonism of histamine-induced calcium mobilization in HeLa cells. HeLa cells were pre-incubated overnight with mepyramine **(A)**, levocetirizine **(B)**, doxepin **(C)**, or olopatadine **(D)**. The next day calcium mobilization was measured upon injection of histamine. Representative graphs are shown of ≥3 experiments with mean ± SD of duplicate peak responses (*n* = 2) normalized to the E_max_ of histamine.

Pre-incubated long-residence-time antagonists can display insurmountable antagonism of receptor signaling if agonist-induced responses are measured before the establishment of a binding equilibrium between agonist, antagonist and the receptor (Kenakin et al., 2006). The slow dissociation of the antagonist from the receptor will reduce the number of receptors available for agonist-induced activation, which is reflected by a decreased E_max_ that might be preceded by an initial rightward shift of the dose response curve in the presence of a receptor reserve (Kenakin et al., 2006; Riddy et al., 2015). It was therefore tested whether insurmountable antagonism of histamine-induced calcium mobilization could be observed when cells were pre-incubated with the antihistamines mepyramine, doxepin, levocetirizine, and olopatadine. This series of H_1_R antagonists reflects a wide range in ligand residence times at the H_1_R (**Table 2**). HeLa cells were pre-incubated with various concentrations of antagonists overnight and cells were subsequently stimulated with increasing concentrations histamine (**Figure 4**). For all four inhibitors the maximal histamine-induced response in HeLa cells was reduced compared to vehicle treated cells. For mepyramine, the H_1_R antagonist with a short residence time at the H_1_R, some decrease in the E_max_ was observed next to a rightward shift of the histamine dose-response curves (**Figure 4A**). For doxepin (**Figure 4B**), levocetirizine (**Figure 4C**), and olopatadine (**Figure 4D**), a full insurmountable inhibition of the histamine-induced calcium mobilization was observed. The insurmountable antagonism was marked by a clear drop in the E_max_ for each subsequent increase in antagonist concentration up to a (near) complete block of any histamine-induced effect at the highest concentrations. These data are in line with the expectations for H_1_R antagonists with a long residence time, but this assay format does not easily allow one to obtain quantitative data on the kinetic properties of the tested H_1_R antagonists. Moreover, the insurmountable antagonism by doxepin could not be differentiated from the insurmountable antagonism of levocetirizine and olopatadine, despite the 6 to 8-fold difference in residence time that was measured in radioligand binding experiments (**Table 2**).

**FIGURE 5:**
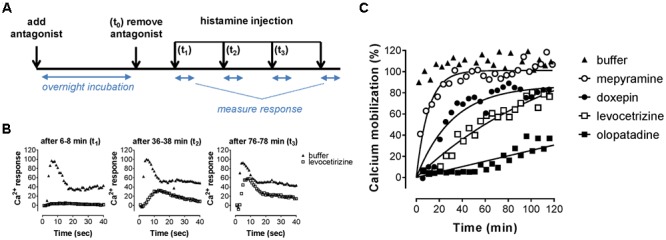
Recovery rate of histamine-induced calcium mobilization upon dissociation of antihistamines from the H_1_R. **(A)** HeLa cells were pre-incubated overnight with 10x K_i_ concentrations of the respective antihistamine. The next day antihistamine dissociation was induced by two rapid wash steps followed by injection of histamine at the indicated time points and calcium mobilization was measured. The resulting calcium-dependent fluorescent-traces **(B)** were quantified by the peak fluorescence. **(C)** Peak fluorescence was plotted against the time between antagonist removal and histamine injection. Representative graphs are shown of ≥3 experiments, with single measurements per time point. The histamine-induced peak fluorescence was normalized to the average peak fluorescence of buffer pre-treated cells.

An alternative assay format was therefore evaluated in which the relative blockade of the receptor was quantified over time in order to discriminate antihistamines with relatively long drug target engagement times (**Figure 5A**). HeLa cells were incubated overnight with a 10-fold K_i_ concentration of the various antagonists. Cell were subsequently washed and stimulated with 10^-5^ M histamine at different time points to probe the responsiveness of the H_1_R (**Figure 5B**). After pre-incubation and subsequent washout of mepyramine from the HeLa cells, histamine rapidly (within 40 min) induces a similar level of calcium mobilization as observed in cell pre-incubated with only buffer. In contrast, pre-incubation with doxepin resulted in a delayed recovery of the histamine induced calcium mobilization and the recovery of histamine responsiveness was even more delayed in cells pre-incubated with levocetirizine and olopatadine. Since the free receptor population after the wash step dependents on the dissociation of the antagonist, the functional response of histamine was expected to recover with rates that would mirror the *k*_off_ of the tested H_1_R antagonists. The receptor recovery over time after washing away unbound antagonist was fitted to a one-phase association model (Eq. 3; **Figure 5C**). This one-phase exponential model is the same as the dissociation model used for describing drug-target dissociation and was observed to fit the functional response over time reasonably well. As a measure for the absolute time-scale in which recovery rate takes place, the k_rec_ was transformed into the receptor recovery time (RecT) by taking the reciprocal of the k_rec_. This is analogous to the way in which residence time is calculated from the *k*_off_ values for the dissociation of ligand binding. The histamine induced response after olopatadine incubation did not approach a steady-state within the time-frame of the experiment. To make an estimate of the receptor recovery rate for olopatadine, the steady-state was constrained to the average histamine induced calcium levels measured in vehicle treated cells. Additionally, after pre-treatment with levocetirizine the functional H_1_R did not fully recovery within the time frame of the experiment either, but the average steady-state recovery (%), reflected by the Y_max_, suggests proper fitting of the curves. The determined k_rec_, RecT and steady-state recovery was quantified for all antihistamines (**Table 3**). As can be seen, analyses allowed for good discrimination between antihistamines over a 40-fold range in k_rec_ and RecT. However, full recovery of histamine induced calcium mobilization was already obtained after 1 or 2 data points following a pre-incubation with, e.g., VUF14454 and VUF14493. The kinetic resolution of this assay cannot readily discriminate between antihistamines with very fast recovery rates of the receptor responses.

**Table 3:** Recovery rate and maximal recovery of histamine induced calcium mobilization.

	k_rec_^b^ (min^-1^)	RecT^c^ (min)	Steady-state recovery (%)	RT^d^ (min)
Mepyramine	0.12 ± 0.03	10 ± 2	103 ± 1	3.9 ± 0.6
Levocetirizine	0.014 ± 0.003	87 ± 20	104 ± 10	140 ± 20
Doxepin	0.032 ± 0.003	32 ± 3	78 ± 5	22 ± 7
Olopatadine^a^	0.006 ± 0.001	226 ± 60	NA^a^	170 ± 10
Triprolidine	0.06 ± 0.01	19 ± 2	92 ± 4	3.5 ± 0.4
Acrivastine	0.08 ± 0.01	13 ± 2	102 ± 2	15.6 ± 0.9
Desloratadine	0.033 ± 0.001	30 ± 1	83 ± 4	160 ± 50
VUF14544	0.22 ± 0.07	6 ± 2	100 ± 2	0.9 ± 0.2
VUF14454	0.12 ± 0.03	9 ± 3	96 ± 5	1.8 ± 0.3
VUF14506	0.07 ± 0.01	15 ± 1	106 ± 5	22 ± 4
VUF14493	0.24 ± 0.06	5 ± 2	98 ± 1	1.1 ± 0.2

*Average values are shown ±SEM of ≥3 experiments.*

*^a^Maximal signal (i.e., full recovery) was constrained during analysis, to the average histamine-induced response observed for vehicle treated cells.*

*^b^k_*rec*_, observed recovery rate of H_1_*R* activity.*

*^c^RecT, recovery time (1/k_*rec*_).*

*^d^RT, residence time (1/*k*_*off*_). Adapted from **Table 2**.*

**FIGURE 6:**
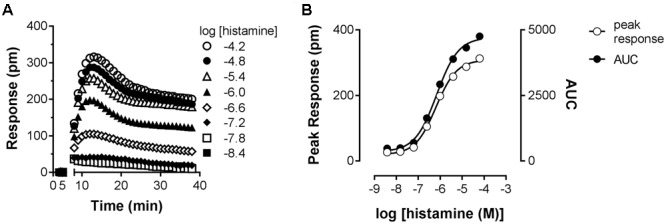
Dynamic mass redistribution (DMR) response induced by histamine. **(A)** A Concentration-dependent DMR response was induced on confluent HeLa cells upon addition of histamine. A positive DMR was observed with a peak response (pm) after approximately 3–5 min. A representative dataset of four experiments is displayed [mean ± SD of triplicate values (*n* = 3)]. **(B)** For comparison, peak response and AUC were determined from the kinetic DMR traces and plotted against the histamine concentration.

As an orthogonal assay to measure the histamine-H_1_R induced response in HeLa cells, DMR was employed. In this assay format cells are grown on RWG biosensors. This allows to detect changes in cellular mass distribution (e.g., translocation of proteins, cytoskeleton rearrangements) close to the biosensor surface (Fang et al., 2006, 2008). DMR was shown to be an effective way to quantify histamine induced signaling mediated by the H_1_R (Lieb et al., 2016). Upon stimulating HeLa cells with increasing concentrations of histamine, DMR response reached a maximum after approximately 5 min (**Figure 6A**), as previously reported for Gα_q_ mediated signaling by other GPCRs (Fang et al., 2008). After fitting the data with a four-parameter sigmoidal fit a pEC_50_ of 6.1 was obtained from both curves with a Hill slope of 0.88 (AUC) and 0.92 (peak response) (**Figure 6B**).

**FIGURE 7:**
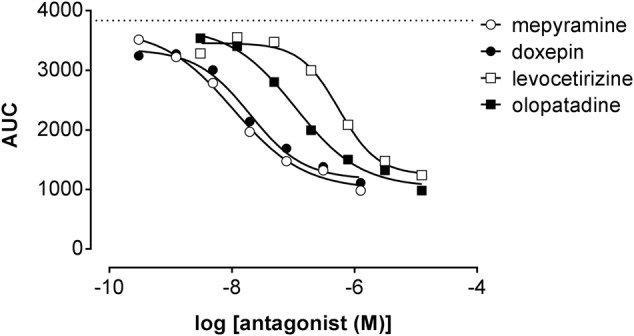
Inhibition of the histamine induced DMR response. HeLa cells were co-incubated with histamine (EC_80_: 2 μM) and increasing concentrations of antihistamines. The AUC was determined in the first 20 min after compound addition and plotted against the respective antihistamine concentration. A dose dependent reduction of the maximal DMR signal (dashed line) was observed for all four antagonists [a representative dataset of two experiments is shown, depicted as mean ± SD of four values (*n* = 4)].

Co-incubating HeLa cells with histamine at a concentration of approximately EC_80_ and increasing concentrations of antihistamines reduced the DMR response concentration-dependently (**Figure 7**) with the following pIC_50_ values determined from AUC analysis (mean ± SD of 2 experiments): mepyramine 8.1 ± 0.2, doxepin 8.0 ± 0.4, olopatadine 7.0 ± 0.1, and levocetirizine 6.3 ± 0.2. It was observed for all inhibitors that the DMR-signal was not fully reduced to background level at the highest concentration. From the full kinetic traces it was observed that the peak response from co-incubated cells stabilized at different levels compared to untreated cells (Supplementary Figure 2). Incubation of cells with antagonists alone resulted in a negligible DMR response, comparable to the background traces (data not shown).

**FIGURE 8:**
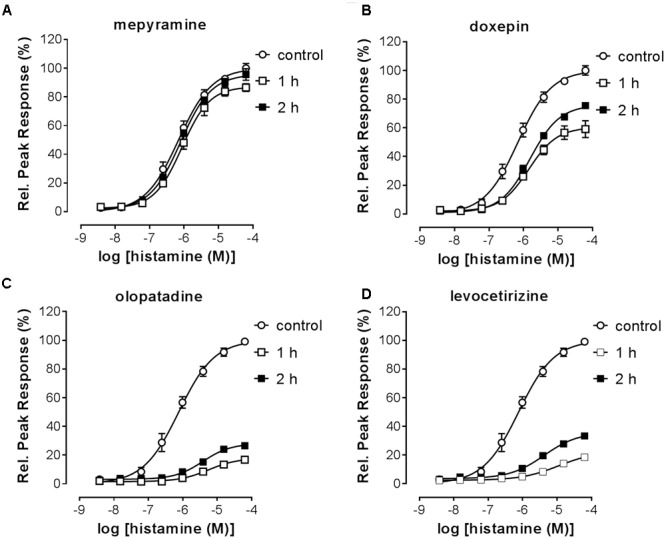
Histamine-induced DMR-response after washout of pre-incubated antihistamines. HeLa cells were incubated overnight in the presence of antagonist (10x K_i_ concentration) or DMSO (control). On the following day the antagonist was removed by washing the cells with assay buffer [HBSS, 20 mM HEPES and 0.5% (v/v) DMSO]. At two different time points (1 h, 2 h) after antagonist washout cells were treated with histamine and the DMR response was recorded over 30 min. Results are shown for the antagonists mepyramine **(A)**, doxepin **(B)**, levocetirizine **(C)** and olopatadine **(D)**. The histamine induced DMR response was normalized against the response of DMSO treated cells. Results from two independent experiments are shown, depicted as mean ± SD of six values (*n* = 6).

The RecT of the histamine-induced DMR response after removal of unbound antihistamines was subsequently quantified. HeLa cells were incubated with a 10x K_i_ concentration of each of the antagonists to ensure high occupancy of the receptor. On the following day cells were washed with assay buffer and antagonists thereby removed. When the DMR signal was detected directly after washing, a drift was observed due to the adaptation of cells to room temperature and DMSO concentration of the assay buffer. The signal stabilized after approximately 1 h (data not shown). Therefore, cells were stimulated with increasing concentrations of histamine earliest 1 h after antagonist washout. The DMR response was recorded for 30 min and baseline corrected for each individual well. The relative suppression of the histamine E_max_ was estimated by comparing the peak response induced at a saturating histamine concentration (63 μM) of cells pre-incubated with or without the respective antihistamine (**Table 4**). The maximal response of histamine was not suppressed by pre-incubation with the fast-dissociating antihistamine mepyramine (**Figure 8A**). In contrast, the slow-dissociating antihistamines levocetirizine and olopatadine (**Figures 8C,D**), suppressed the E_max_ up to 2 h after removing unbound antihistamines. Pre-incubation with the medium-slow dissociating doxepin suppressed E_max_, but to a lower extent than olopatadine and levocetirizine (**Figure 8B**).

**Table 4:** Suppression of the histamine induced DMR-E_max_ after removal of unbound antihistamines.

		1 h after washout		2 h after washout			
	Log [ligand]	E_max_, %	Histamine pEC_50_	E_max_, %	Histamine pEC_50_	k_rec_^a^ (min^-1^)	RecT^b^ (min)
Vehicle control	NA	100 ± 2	6.1 ± 0.1	100 ± 2	6.2 ± 0.1	NA	NA
Mepyramine	–7.5	87 ± 3	6.1 ± 0.1	96 ± 4	6.1 ± 0.1	0.027 ± 0.001	38 ± 1
Levocetirizine	–7.2	19 ± 1	5.0 ± 0.1	33 ± 2	5.4 ± 0.1	0.0030 ± 0.0001	338 ± 9
Doxepin	–8.3	59 ± 6	5.8 ± 0.1	76 ± 1	5.8 ± 0.1	0.011 ± 0.001	87 ± 6
Olopatadine	–8.1	17 ± 2	5.3 ± 0.1	27 ± 2	5.4 ± 0.1	0.0024 ± 0.0005	429 ± 81
Triprolidine	–7.3	72 ± 8	5.6 ± 0.2	75 ± 6	5.6 ± 0.1	NA	ND
Acrivastine	–6.2	93 ± 2	6.1 ± 0.2	101 ± 2	6.3 ± 0.1	0.038 ± 0.001	26 ± 1
Desloratadine	–8.5	53 ± 4	5.4 ± 0.3	63 ± 2	5.4 ± 0.1	0.0090 ± 0.0007	111 ± 8
VUF14454	–7.2	100 ± 3	6.1 ± 0.1	101 ± 2	6.2 ± 0.1	NA	<30
VUF14544	–6.6	99 ± 3	6.1 ± 0.1	103 ± 5	6.1 ± 0.1	NA	<30
VUF14506	–6.7	87 ± 2	6.1 ± 0.1	98 ± 2	6.2 ± 0.1	0.029 ± 0.003	34 ± 3
VUF14493	–7.4	98 ± 3	6.2 ± 0.1	102 ± 3	6.1 ± 0.1	NA	<30

*A dose-dependent DMR response was observed for increasing concentrations histamine as was evaluated 1 and 2 h after washing away unbound antihistamines. The EC_*50*_ was determined as before (**Figure 8**) and the E_*max*_ value was estimated from the response of 10^*-4.2*^ M histamine and normalized to the E_*max*_ of vehicle treated cells. Values represent mean and SD of *n* = 6 measured over two different days.*

*^a^k_*rec*_, observed recovery rate of H_*1*_R activity.*

*^b^RecT, recovery time (1/k_*rec*_).*

Insurmountable antagonism was pronounced for olopatadine, levocetirizine, desloratadine, doxepin, acrivastine and VUF14506. Triprolidine also showed insurmountable antagonism, but this was the same at 1 and 2 h after washing away unbound antagonist, indicating that binding equilibrium had been reached. As such, the observed level of insurmountable antagonism is most likely not caused by prolonged drug-target occupancy, but might be due to a yet unidentified mechanism of action of triprolidine affecting the DMR response in HeLa cells. The receptor recovery rate after preincubation with each of the antihistamines was estimated from the relative effect of a saturating concentration histamine, obtained at 1 and 2 h after removal of unbound antihistamines (see section “Materials and Methods”).

**FIGURE 9:**
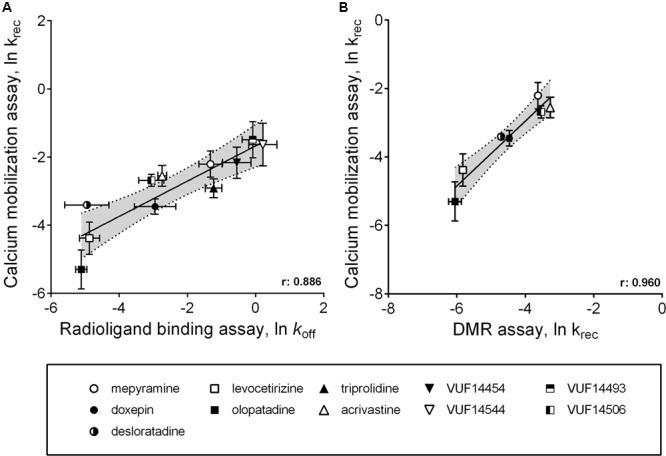
Comparison between ligand dissociation from the H_1_R and the recovery of histamine induced H_1_R signaling after inhibition of the receptor. **(A)** Correlations are depicted between the ln *k*_off_, as determined by radioligand binding experiments, against the ln k_rec_, which is the recovery of histamine induced H_1_R activation after removal of unbound antihistamines. The ln k_rec_ was determined from the calcium mobilization experiments (**Figure 5**). **(B)** An additional correlation was shown for the ln k_rec_ determined by both the calcium mobilization experiments and from DMR experiments (**Figure 8**).

A comparison between the receptor recovery rates and kinetic dissociation rate constants of the set of antihistamines from **Tables 2–4** are depicted in **Figure 9** and Supplementary Table 1. The ln *k*_off_ of the antihistamines correlated with the ln k_rec_ of the histamine-induced DMR (not shown) and calcium mobilization upon washout of these antihistamines (**Figure 9A**). Moreover, the ln k_rec_ as determined by both methods correlated to each other also very well (**Figure 9B**). Although the receptor recovery times and residence times seem to be related, the absolute values of the various metrics are not in the same range. In general, recovery time of the DMR response seem to take longer than for the calcium mobilization (**Tables 3, 4** and Supplementary Table 1).

## Discussion

Drug-target residence time has received a lot of attention over the last decade and has now been suggested as a way to better predict drug-efficacy *in vivo* in an early stage of drug development (Swinney, 2004; Copeland et al., 2006; Lu and Tonge, 2010; Guo et al., 2014; Hoffmann et al., 2015). It has been postulated that a long drug-target residence time can prolong the *in vivo* effects of such drugs compared to drugs with a shorter residence time. Moreover long-residence antagonists might exert insurmountable antagonism of the receptor, which is independent on the concentration agonist and can therefore be much stronger than competitive antagonism (Kenakin et al., 2006; Vauquelin et al., 2012). However, whether a long drug-target residence time is beneficial for the *in vivo* efficacy, is dependent on the specific biology of the drug-target (Schuetz et al., 2017). For example, a long drug-target residence time only leads to a prolonged receptor occupancy *in vivo* if the receptor turnover is low (Hong et al., 2008; Bradshaw et al., 2015). Moreover, an insurmountable mode of antagonism is also highly dependent on the cellular context, like e.g., the expression level of the target-receptor (Kenakin et al., 2006). For several GPCR-targets the importance of a prolonged drug-occupancy has however, been advocated and the residence time of GPCR ligands are therefore currently explored as a way to increase the therapeutic window (Guo et al., 2014).

The determination of drug-target residence time for GPCR-ligands is usually performed by radioligand binding experiments, or more recently, by fluorescent ligand binding experiments (Schiele et al., 2015; Bosma et al., 2016; Schuetz et al., 2017). The residence time of unlabeled ligands can be quantified by measuring their competitive effects on the probe binding-kinetics, using the Motulsky–Mahan model (Motulsky and Mahan, 1984). Although the residence time values obtained in these experiments describe the kinetics of GPCR binding at the molecular level, these parameters do not necessarily directly reflect the functional effects in a physiological relevant setting. In order to bridge the gap between GPCR binding studies on membrane preparations and the *in vivo* evaluation of the action of long residence time GPCR ligands, we evaluated the kinetics of GPCR antagonism for a set of H_1_R antagonists with different ligand binding kinetics in two cell-based assays. The potential success of such a strategy is exemplified for the Neurokinin 1 Receptor, for which it was shown that prolonged antagonism after a washout of unbound antagonists, corresponded with the respective therapeutic window of these antagonists *in vivo* (Lindström et al., 2007). Moreover, the functional effects observed for the set of H_1_R antagonists were compared to the observed residence times, as determined in [^3^H]mepyramine competition binding assays.

In this study, we employed two GPCR assays that can be performed without altering the genetic cellular context and which can be broadly applied to measure GPCR signaling. The calcium mobilization assay has been widely used to measure signaling of GPCRs that couple to the Gα_q/11_ protein, but also GPCRs that couple to Gα_i_ subunits can still induce a calcium mobilization response via released βγ-subunits (Charlton and Vauquelin, 2010). The DMR-response relies on the morphological changes of the cell, which has been linked to all canonical GPCR-signaling cascades (Schröder et al., 2010). HeLa cells, endogenously expressing the H_1_R at low, but physiological relevant densities, were used in both assays.

Stimulation of HeLa cells with histamine induced both a robust calcium-mobilization (**Figure 3**), as well as DMR-response (**Figure 6**) in the HeLa cells with similar potencies (pEC_50_ 6.1–6.2). The rise in intracellular calcium concentrations was clearly more rapid (within seconds, **Figure 3**), than the induction of DMR response (within 5–10 min, **Figure 6**), in line with previous reports (Smit et al., 1992; Lieb et al., 2016). The calcium response was effectively antagonized by the tested H_1_R antagonists, but whereas co-incubation with the short residence time antagonist mepyramine resulted in rightward shifts of the histamine dose response curves with some depression of the maximal response, the long residence time antagonists like doxepin, levocetirizine, and olopatadine (**Figure 4**) showed substantial insurmountable antagonism. The slow dissociation of these long residence time antagonists from the H_1_R strongly suggests a temporal insurmountable mode of antagonism by engaging to the orthosteric binding site. An allosteric binding mechanism might theoretically also explain the depression of the maximal histamine response (Kenakin et al., 2006). Yet, this seems unlikely in view of the overlapping binding sites of the tested antagonists and histamine, as experimentally determined by x-ray crystallography (Shimamura et al., 2011), site-directed mutagenesis studies (Nonaka et al., 1998; Wieland et al., 1999; Gillard et al., 2002; Cordova-Sintjago et al., 2012) and molecular modeling experiments (Shimamura et al., 2011; Cordova-Sintjago et al., 2012; Kooistra et al., 2013). Recently, insurmountable antagonism of the muscarinic acetylcholine receptor and orexin-2 receptor was used to calculate *k*_off_ rate constants of antagonists (Mould et al., 2014; Riddy et al., 2015). The observed insurmountable antagonism of the histamine induced calcium response (**Figure 4**) could potentially also be used to discriminate antihistamines based on their relative *k*_off_ at the H_1_R. However, the used hemi-equilibrium model (Kenakin et al., 2006) is only applicable in the case of a partial insurmountable effect, as was observed for mepyramine at the highest concentrations (**Figure 4A**). Since saturating concentrations doxepin (**Figure 4B**), levocetirizine (**Figure 4C**), and olopatadine (**Figure 4D**) completely block the response to histamine, it is not possible to distinguish these antihistamines by this means.

As alternative assay-format, the functional recovery time (RecT) of the H_1_R responsiveness was measured after washing away pre-bound antihistamines. In this study, 40-fold differences in the RecT were observed for the tested H_1_R antihistamines, including fivefold (DMR) to eightfold (calcium mobilization) differences in RecT between doxepin, levocetirizine, and olopatadine. Hence, the functional recovery time allows the discrimination of antihistamines with a broad range of H_1_R residence times. For both the calcium mobilization and the DMR-assay, a similar trend in the k_rec_ of the H_1_R was obtained after pre-incubating with the various antihistamines (**Figure 9B**). Moreover, the measured k_rec_ values nicely correlated with the *k*_off_ values of the respective H_1_R antihistamines, as measured by [^3^H]mepyramine competition binding kinetics (**Figure 9A**). Our data suggests that the drug-target residence time indeed prolongs the antagonism by H_1_R-antihistamines following washout and hence a rapid and strong reduction of its free concentration ligand. Moreover, our observations are also in line with the prolonged therapeutic window *in vivo* that has been described for some long residence time antihistamines (Schoepke et al., 2013).

The presented methods effectively determine the length of receptor antagonism using a functional readout that is broadly applicable to GPCR research. The measured non-equilibrium antagonism (RecT), which e.g., reflects the level of insurmountable antagonism (**Figure 4**), is proposed to affect the *in vivo* efficacy of drugs (Kapur and Seeman, 2001; Vauquelin et al., 2012). Insurmountable antagonism is described to be affected by the transduction coefficient of the functional receptor response and is therefore dependent on the cellular context (Kenakin et al., 2006). Since the described methods are applicable to cell lines endogenously expressing the target receptor, RecT does not only reflect the relative residence time, it has the potential to reflect which antagonists have a long enough residence time to impose prolonged non-equilibrium antagonism. Recently, a different functional readout of the H_1_R was employed to measure the relative residence time of antihistamines on the H_1_R (Bosma et al., 2016). This BRET based β-arrestin2 recruitment assay allows a continuous readout of the H_1_R response that was stable in time, improving therefore the throughput in which the relative residence time was determined. As a downside, this method requires genetic manipulation of cells by introducing a tagged H_1_R and tagged β-arrestin2, resulting therefore in, e.g., non-physiologically relevant expression levels.

One limitation of the washout experiments is the possibility that unbound antihistamines are not completely removed by the wash step. An incomplete recovery of receptor signaling was, e.g., observed after pre-incubation with doxepin and desloratadine (**Figure 5B** and **Table 3**). During the washout, only the unbound ligands are removed and the dissociation of H_1_R-bound ligands might provide enough ligands after the initial washout to result in residual receptor occupancy. However, this would only happen if the binding affinity (pK_i_) is high enough to allow binding at these low ligand concentrations, especially as HeLa cells express low numbers of H_1_Rs. Interestingly, doxepin and desloratadine have the highest pK_i_ values of all the antihistamines that were tested in this study (**Table 2**). Another confounding factor in the assay might be the potential partitioning of H_1_R ligands in the cell or membranes, resulting in ligands accumulation and H_1_R rebinding after washout of free ligands. Yet, this issue has also been described to affect the determination of residence times via the kinetic competition binding method (Sykes et al., 2014).

The kinetic information in the DMR washout experiments was low and to prevent over-interpretation of the data, the k_rec_ values were only estimated when there was a significant difference in the histamine induced DMR response between 1 and 2 h after washout of the antihistamines. As a drawback, this could exclude compounds which have a very low k_rec_ value, but this seems unlikely for all ligands that were excluded here, since in all cases a >70% recovery of the histamine induced effect was observed already 1 h after the wash step. For the evaluated H_1_R ligands the calculated k_rec_ values obtained in the DRM assay correlate well with the values obtained in the orthogonal calcium-mobilization assay, despite the fact that the kinetic resolution of the measurements was much lower.

## Conclusion

In this study we describe two orthogonal, functional kinetic assays for GPCR ligands, that can be performed with cells that have endogenous expression of the receptor without the need for any genetic manipulation. The obtained RecT values for H_1_R antagonists correlate well with their H_1_R residence times as determined by radioligand binding. Hence, the use of RecT as a drug metric for the kinetics of antagonism might be valuable to steer the lead optimization of GPCR, as the described generic assays can potentially be used to measure the kinetics of antagonism of ligands for a large variety of GPCRs. Moreover, the assays can additionally be employed with clinically relevant cell lines (e.g., primary cells).

## Author Contributions

Participated in research design: RB, GW, PG, HV, SG, and RL. Conducted experiments: RB, GW, and IJ. Performed data analysis: RB, GW, LV, and IJ. Wrote or contributed to the writing of the manuscript: RB, GW, LV, IJ, PG, HV, SG, and RL.

## Conflict of Interest Statement

The authors declare that the research was conducted in the absence of any commercial or financial relationships that could be construed as a potential conflict of interest.
