# Supplementary material for: The Target Residence Time of Antihistamines Determines Their Antagonism of the G Protein-Coupled Histamine H1 Receptor
Source: Front Pharmacol. 2017 Sep 25;8:667. doi: 10.3389/fphar.2017.00667 (PMC5627017; doi:10.3389/fphar.2017.00667)
Supplement: Supplementary file 1 [file Data_Sheet_1.docx]

Supplementary Material

The target residence time of antihistamines determines their functional antagonism of the G protein-coupled histamine H1 receptor

**Reggie Bosma^1^, Gesa Witt^2^, Lea Vaas^2^, Ivana Josimovic^1^, Philip Gribbon^2^, Henry F. Vischer^1^, Sheraz Gul^2^, Rob Leurs^1^***

^1^Amsterdam Institute for Molecules, Medicines and Systems (AIMMS), Division of Medicinal Chemistry, Faculty of Science, Vrije Universiteit Amsterdam, De Boelelaan 1108, 1081 HZ Amsterdam, The Netherlands.

^2^Fraunhofer Institute for Molecular Biology and Applied Ecology Screening Port, D-22525 Hamburg, Germany.

*** Correspondence:**Rob Leurs
[r.leurs@vu.nl](mailto:r.leurs@vu.nl)

# Supplementary Figures and Tables

## Supplementary Figures


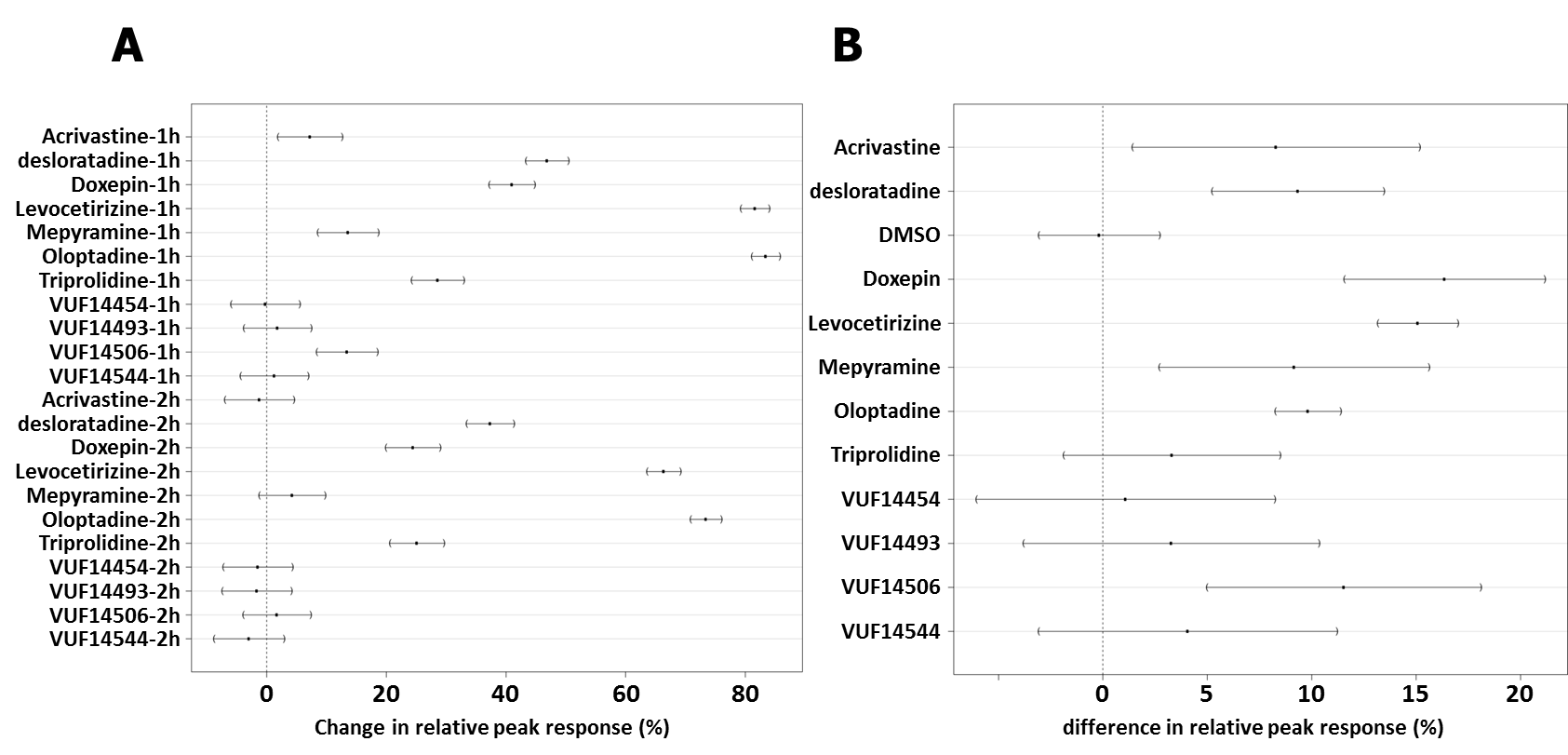


**Supplementary Figure 1-Statistical differences in DMR peak responses.** Generalized linear model with Gamma family and identity link (Additive arithmetic mean model) of the form *Relative.peak.response.. ~ treatment.combi + Experimental.Repeat* was build. The outcome variable is highly skewed, but always positive and the effects of treatment-wash-out-time and experimental repetitions are assumed to be additive on the original scale. Multiple comparisons of means with type I error rate of 5% were calculated for comparing the effect of antihistamines in comparison to the DMSO-control for both wash-out-times (**A**) and the effect of the wash-out-time for each respective antihistamine (**B**). All computations were performed in R (R Development Core Team, 2016) and MCPs were computed using the functionality provided by the add-on package multcomp (Hothorn *et al.*, 2008) R Core Team (2016).

*References:*

Hothorn T, Bretz F, and Westfall P (2008) Simultaneous Inference in General Parametric Models. *Biometrical J* **50**:346–363.

R Development Core Team (2016) R: A Language and Environment for Statistical Computing. *R Found Stat Comput Vienna Austria* **0**:{ISBN} 3-900051-07-0.


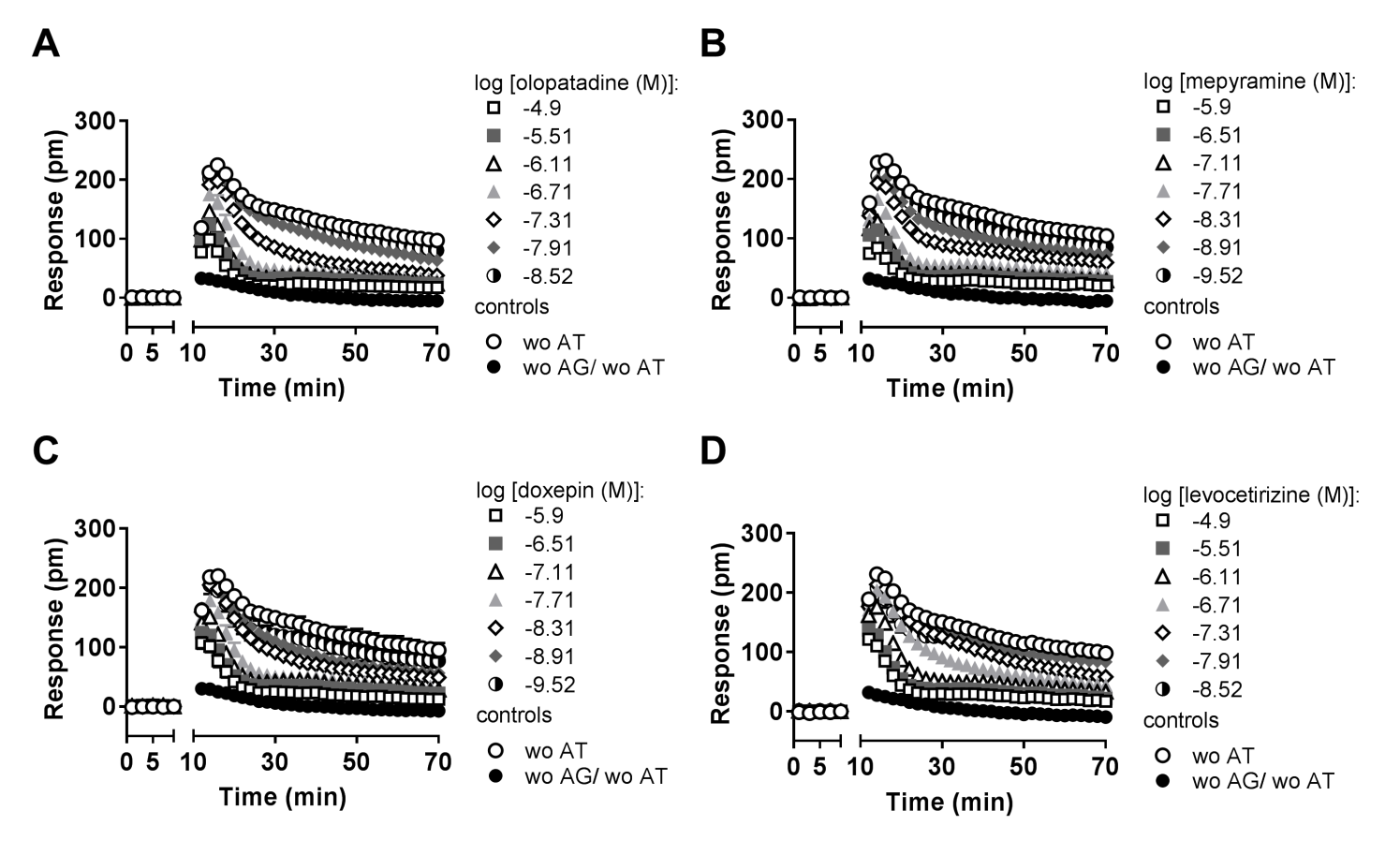


Supplementary Figure 2-DMR traces of agonist/antagonist co-incubation assay. HeLa cells were incubated in parallel with histamine and increasing concentrations of olopatadine (A), mepyramine (B), doxepin (C) or levocetirizine (D). An additional control condition was included without antagonist (wo AT) and a condition without either agonist or antagonist (wo AG/wo AT). Area under the curve was determined in the first 20 min after compound addition and plotted against the antagonist concentration to determine an apparent IC_50_ (Fig 7 in the manuscript).

Supplementary Table 1 - Structures of H_1_R antihistamines with binding affinities, residence times and recovery times.

| # | ligand | structure | pK_i_ | pK_d,calc_ | RT (min) | RecT (min)  ca^2+^ mob. | RecT (min)  DMR |
| --- | --- | --- | --- | --- | --- | --- | --- |
| 1 | mepyramine |  | 8.5 ± 0.0 | 8.8 ± 0.0 | 3.9 ± 0.6 | 10 ± 2 | 38 ± 1 |
| 2 | levocetirizine |  | 8.1 ± 0.1 | 8.2 ± 0.1 | 140 ± 20 | 87 ± 20 | 338 ± 9 |
| 3 | doxepin |  | 9.3 ± 0.1 | 9.1 ± 0.1 | 22 ± 7 | 32 ± 3 | 87 ± 6 |
| 4 | olopatadine |  | 9.1 ± 0.0 | 8.5 ± 0.0 | 170 ± 10 | 226 ± 60 | 429 ± 81 |
| 5 | triprolidine |  | 8.3 ± 0.1 | 8.1 ± 0.1 | 3.5 ± 0.4 | 19 ± 2 | ND |
| 6 | acrivastine |  | 7.2 ± 0.0 | 7.0 ± 0.0 | 15.6 ± 0.9 | 13 ± 2 | 26 ± 1 |
| 7 | desloratadine |  | 9.1 ± 0.1 | 9.5 ± 0.0 | 160 ± 50 | 30 ± 1 | 111 ± 8 |
| 8 | VUF14544 |  | 7.6 ± 0.1 | 7.8 ± 0.1 | 0.9 ± 0.2 | 6 ± 2 | <30 |
| 9 | VUF14454 |  | 8.2 ± 0.1 | 8.6 ± 0.0 | 1.8 ± 0.3 | 9 ± 3 | <30 |
| 10 | VUF14506 |  | 7.7 ± 0.1 | 7.9 ± 0.0 | 22 ± 4 | 15 ± 1 | 34 ± 3 |
| 11 | VUF14493 |  | 8.3 ± 0.0 | 8.4 ± 0.1 | 1.1 ± 0.2 | 5 ± 2 | <30 |
